# Adhesive small bowel obstruction in a 3-year-old child with history of necrotizing enterocolitis: a case report

**DOI:** 10.3389/fsurg.2026.1935396

**Published:** 2026-10-20

**Authors:** Ghadah Alsaleh, Khaled Bajaeifer, Mohammed Alawi Alsakkaf

**Affiliations:** 1 Global Center for Mass Gathering Medicine, Ministry of Health, Riyadh, Saudi Arabia; 2 Emergency Medicine Department, Neom Hospital, Tabuk, Saudi Arabia; 3 General Surgery Department, Neom Hospital, Tabuk, Saudi Arabia

**Keywords:** adenovirus, adhesive small bowel obstruction, case report, Entamoeba histolytica, ESBL Escherichia coli, necrotizing enterocolitis, postoperative adhesions

## Abstract

**Background and objectives:**

Adhesive small bowel obstruction (ASBO) is a serious complication following abdominal surgery in children, yet its presenting symptoms often mimic non-surgical conditions such as viral mesenteric adenitis and functional constipation. This diagnostic challenge is particularly acute in young children with a history of neonatal intestinal surgery who cannot reliably communicate their symptoms. This case report describes a former extreme preterm infant with surgical history of necrotizing enterocolitis (NEC) who presented with acute abdominal pain and was found to have complete mechanical small bowel obstruction due to postoperative adhesions. The objective is to highlight the diagnostic pitfalls in this population and to describe an unusual postoperative course complicated by three concurrent infections.

**Case presentation:**

A 3-year-7-month-old former preterm girl (23 weeks gestation) with a history of necrotizing enterocolitis (NEC) requiring jejunal resection and stoma closure presented with one day of colicky abdominal pain, vomiting, and constipation. Initial ultrasound showed reactive mesenteric lymph nodes and fecal loading, suggesting mesenteric adenitis and constipation. However, given her surgical history, she was admitted for observation. Abdominal x-ray revealed small bowel dilatation with air-fluid levels, and contrast-enhanced CT confirmed complete mechanical small bowel obstruction with pneumatosis intestinalis. She underwent emergent laparotomy, which revealed extensive adhesive bands causing obstruction without volvulus, and adhesiolysis was performed without complication. On postoperative day 17, she developed a one-day fever. Workup revealed three concurrent infections: ESBL-producing Escherichia coli urinary tract infection, adenovirus, and Entamoeba histolytica/dispar. She was treated with intravenous meropenem and metronidazole and recovered fully.

**Conclusion:**

Children with prior intestinal surgery can present with acute abdominal pain that mimics non-surgical conditions such as mesenteric adenitis and constipation while having a complete adhesive small bowel obstruction requiring emergent surgery. Normal or equivocal ultrasound findings do not exclude this diagnosis, and a low threshold for CT is essential. Postoperative fever in such patients warrants a broad infectious workup, as multiple concurrent pathogens may coexist.

## Introduction

Adhesive small bowel obstruction (ASBO)is a common and potentially life-threatening complication following abdominal surgery in children (1). The formation of fibrous bands between loops of intestine, known as adhesions, occurs in virtually all patients after peritoneal injury, though only a subset develops clinically significant obstruction (2). Adhesions typically form within the first month after surgery as part of the normal peritoneal healing response, but their pathological progression into symptomatic obstruction can take months or even years to manifest, and obstruction can occur suddenly without prior warning symptoms (3). In the pediatric population, the incidence of adhesive obstruction following laparotomy ranges from 5% to 25%, with the highest risk observed in neonates who undergo intestinal surgery for conditions such as necrotizing enterocolitis (NEC), intestinal atresia, or gastroschisis (4, 5).

The diagnosis of Adhesive small bowel obstruction in young children presents unique challenges. Presenting symptoms, abdominal pain, vomiting, constipation, and abdominal distension, are non-specific and overlap substantially with far more common non-surgical conditions such as viral gastroenteritis, mesenteric adenitis, and functional constipation (2). Young children, particularly those of preschool age, cannot reliably articulate the quality, location, or severity of their pain, nor can they provide a detailed history of associated symptoms. This reliance on parental observation and physical examination alone introduces significant uncertainty into the diagnostic process (6). In a child with prior laparotomy, the consequences of missing an adhesive obstruction are severe, as delayed diagnosis can lead to bowel ischemia, perforation, peritonitis, sepsis, and death (7). This diagnostic dilemma is further complicated by the radiation exposure associated with computed tomography (CT) in young children and the difficulty of performing serial abdominal examinations in a distressed or uncooperative child (7, 8).

This case is noteworthy because it involves a child with a complex surgical history in whom the initial presentation mimicked non-surgical conditions, creating a diagnostic challenge with potentially serious consequences. Additionally, the postoperative course revealed an unusual combination of concurrent infections that are rarely described together in the literature. This report aims to describe the successful management of this complex case and to provide practical lessons for clinicians evaluating children with prior abdominal surgery who present with acute abdominal pain.

## Case presentation

### Patient history & clinical findings

A 3-year-7-month-old girl presented to the emergency department with a one-hour history of severe diffuse abdominal pain. According to her mother, the child had been constipated for the preceding two days but had eaten her breakfast normally and then began complaining of intense pain. The mother observed that the child attempted to have a bowel movement without success, and the pain worsened with straining. The child was noted to be curling her body in response to the pain, a posture consistent with significant intra-abdominal discomfort. There was no associated vomiting at the time of initial presentation, no fever, and no urinary symptoms. The initial emergency department examination revealed a well-appearing, alert child with stable vital signs, but there was notable abdominal guarding such that she cried when the abdomen was palpated. Based on these findings, the decision was made to transfer the patient via ambulance to another hospital for further evaluation.

Upon arrival at the hospital later that same day, the history was further elaborated. The child had experienced central to epigastric colicky abdominal pain that was non-radiating and had been ongoing for approximately 24 h. One episode of vomiting had occurred. The mother reported that the child had not passed stool on the day of presentation and had decreased oral intake. There was a history of runny nose approximately one week prior to presentation, suggesting a possible preceding viral illness. Review of systems was otherwise negative for diarrhea and urinary frequency or dysuria. The patient's past medical history was remarkable for prematurity at 23 weeks gestation, with a birth weight of 435 grams, complicated by respiratory distress syndrome, persistent pulmonary hypertension, recurrent left pneumothorax, bronchopulmonary dysplasia, and patent ductus arteriosus that closed with paracetamol therapy. Most significantly, she developed NEC on day 52 of life requiring laparotomy with resection of 20 cm of jejunum and creation of a jejunostomy. By postoperative day 17, the stoma was successfully closed and she tolerated full oral feeding. Additional past medical history included anemia of prematurity which was resolved, borderline thyroid function tests that normalized, periventricular leukomalacia on neonatal brain ultrasound, mild speech delay, and no evidence of adrenal insufficiency. She had no hospitalizations since NICU discharge, and she was not on any regular medications at the time of presentation. Allergies included moderate reactions to beef and cow's milk, both manifesting as itching.

Physical examination on admission to the hospital revealed an ill-appearing child in apparent pain with signs of mild dehydration. Vital signs were stable with heart rate, respiratory rate, blood pressure, and oxygen saturation within normal limits for age. Throat examination showed congestion. Chest auscultation revealed clear breath sounds bilaterally with no wheezes or crackles. Abdominal examination was notable for soft but tender abdomen, with tenderness localized to the central and lower abdomen. Importantly, there was no right iliac fossa tenderness, no guarding, no rebound tenderness, and bowel sounds were present. However, given the child's high-risk surgical history, the clinical team proceeded with admission for observation and serial abdominal examinations rather than discharge.

### Diagnostic assessment & management

The initial diagnostic workup included laboratory studies and abdominal ultrasound. Serum C-reactive protein was mildly elevated at 7 mg/L, a non-specific finding that could be consistent with either an inflammatory or infectious process. A complete blood count obtained on the day of admission showed a white blood cell count of 3.94 × 10^3^/*μ*L, which is below the normal range of for age. The differential revealed 65.5% neutrophils, 28.2% lymphocytes (low), and 6.3% monocytes, with eosinophils and basophils at 0%. Hemoglobin was 13.2 g/dL (within normal range) and platelet count was 267 × 10^3^/μL (normal). Subsequent CRP monitoring showed a progressive rise to 42.2 mg/L, 126 mg/L, and 106 mg/L.

Abdominal ultrasound performed on the day of admission was limited due to a gassy abdomen and fecal material, which are well-recognized factors that reduce the diagnostic accuracy of ultrasound for bowel obstruction, but it revealed multiple hypoechoic periumbilical and right lower quadrant lymph nodes, the largest measuring 1.8 cm, which were interpreted as likely reactive. The appendix was not visualized. There was no free fluid or localized collection. The liver, gallbladder, pancreas, spleen, and kidneys were all unremarkable. The bladder was underfilled but appeared unremarkable. Based on these ultrasound findings combined with the history of constipation, the initial assessment was acute abdominal pain likely secondary to mesenteric adenitis and constipation with fecal loading. However, the clinical team explicitly noted that given the patient's past surgical history of NEC with bowel resection, it was necessary to exclude an evolving surgical abdomen or pancreatitis.

The diagnostic turning point occurred when abdominal x-rays (supine and erect views) were obtained. The radiographs demonstrated few air-fluid levels in the mid abdomen and pelvis associated with small bowel dilatation measuring up to 3.5 cm in diameter. Additionally, a “peak sign” and abrupt obstruction of mid-abdomen small bowel loops were noted, findings highly suggestive of bowel obstruction, possibly due to volvulus. No pneumoperitoneum was observed. In response to these concerning findings, the surgical team was consulted, and a contrast-enhanced CT of the abdomen and pelvis with intravenous contrast was performed emergently. The CT scan confirmed mechanical complete small bowel obstruction. Small bowel loops were dilated to 3 cm in maximum diameter, and large bowel loops were dilated to 3.7 cm. More concerningly, the scan demonstrated wall edema of the rectum and sigmoid loops with air within the bowel wall, pneumatosis intestinalis, causing proximal dilatation of the large bowel. Rotating superior mesenteric vessels at the mid jejunal level and an adhesion band within jejunal loops with areas of narrowing were seen, suggestive of either volvulus or post-surgical adhesions. The impression was mechanical complete small bowel obstruction due to volvulus or adhesions, with pneumatosis intestinalis of the rectosigmoid colon. The surgical team was immediately notified.

Following confirmation of complete small bowel obstruction, the patient was kept NPO (nil per os), intravenous fluids (D5% normal saline at 50 mL/hr) were continued, and she was prepared for emergency surgery. A chest x-ray obtained prior to surgery showed bilateral subtle granular opacities with right-sided predominance and mild pleural effusion, findings suggestive of an inflammatory or infectious process, though the patient was afebrile at that time. Intraoperative findings included extensive small bowel adhesions and multiple adhesive bands causing obstruction (Figure 1). The surgeon performed extensive small bowel adhesiolysis, excision of multiple bands, and an incidental appendectomy. The procedure was completed without complication and with minimal blood loss as documented by the operating surgeon. There was no volvulus identified intraoperatively; the obstruction was purely adhesive in nature. The patient tolerated the procedure well and was transferred to the surgical ward for postoperative care.

**Figure 1:**
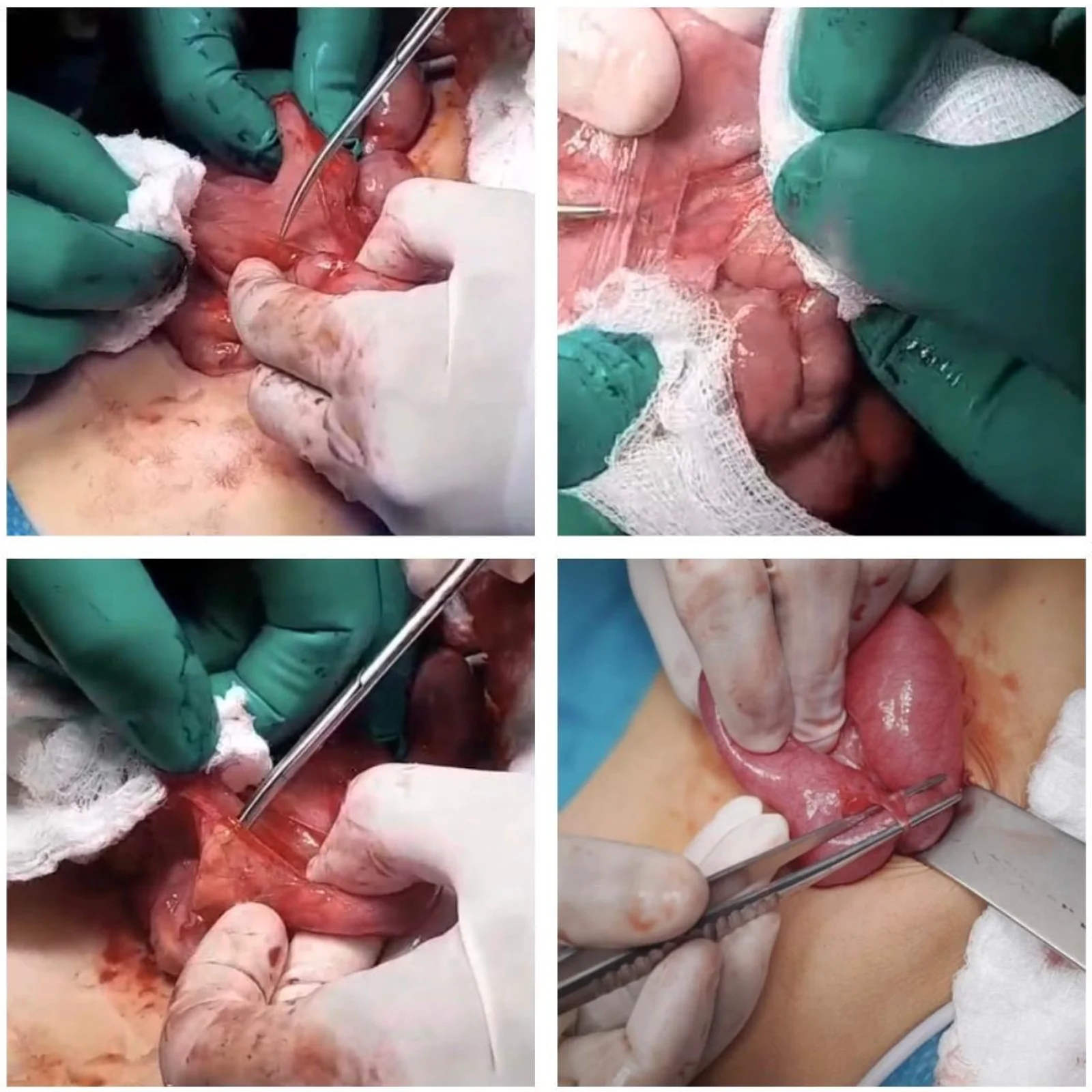
Adhesiolysis due to severe small intestine adhesions with zones of complete obstruction.

### Outcome & follow-up

On the first postoperative day, the patient was hemodynamically stable and appeared well, not in pain or distress. Her vital signs remained within acceptable parameters. The abdomen was soft but not fully examinable due to the fresh surgical site. She was maintained NPO with intravenous D5% normal saline and received regular paracetamol 170 mg intravenously every six hours as needed for pain control. The surgical team ordered monitoring of complete blood count and electrolytes, and they planned to follow the pediatric surgery plan closely.

By postoperative day three, the patient showed significant clinical improvement. Abdominal distention had decreased, she had not vomited, she was ambulating, and she had been started on an oral soft diet. She was passing urine freely and regularly, and she had passed a moderate amount of more watery stool once during the night and once in the morning. She was receiving oral high-energy nutritional supplement, oral rehydration solution, intravenous fluids with potassium chloride, and oral macrogol for constipation prevention. The abdominal examination at this time showed a mobile abdomen with respiration, very mild distension, a clean dry and intact surgical wound, a soft abdomen, and audible bowel sounds on auscultation.

The postoperative course was complicated by a one-day fever along with persistent abdominal distension that had been noted since the surgery. The viral respiratory panel returned positive for adenovirus. Urine culture grew extended-spectrum beta-lactamase (ESBL)-producing Escherichia coli, establishing the diagnosis of a urinary tract infection with a multidrug-resistant organism. Stool analysis revealed the presence of Entamoeba histolytica/dispar cysts and trophozoites, indicating possible intestinal amebiasis. The stool analysis also showed greenish, mucoid consistency with 7 pus cells and 2 + mucus. By the time these results were available, the patient's fever had resolved spontaneously. She had started oral intake without vomiting and had passed stool. Her vital signs were stable. The physical examination documented a soft abdomen with distention, normal bowel sounds, and no guarding.

The treatment plan, as approved by the pediatric consultant, consisted of intravenous meropenem at 20 mg/kg/dose every 8 h (215 mg three times daily) for the ESBL E. coli urinary tract infection, combined with intravenous metronidazole for a total of 5 days to cover both anaerobic bacteria and the Entamoeba infection. The patient was encouraged to take oral intake gradually as tolerated. No specific antiviral therapy was administered for adenovirus. By the time of discharge, the patient had recovered fully, tolerating an oral diet, passing stool regularly, and afebrile off antibiotics. She was discharged home with outpatient follow-up arranged with both the surgery and pediatrics teams.

## Discussion

This case describes a former preterm infant with surgical history of necrotizing enterocolitis who presented with acute abdominal pain mimicking mesenteric adenitis and constipation, but was found to have complete ASBO requiring adhesiolysis, complicated postoperatively by three concurrent infections. Each element of this case offers an educational value that aligns with and expands upon the existing evidence-based guidelines for ASBO management (8).

A key learning point from this case concerns the diagnostic challenge of distinguishing ASBO from non-surgical causes of abdominal pain in children with a history of abdominal surgery. The Bologna guidelines of the World Society of Emergency Surgery emphasize that history taking and physical examination alone have low sensitivity for detecting bowel strangulation and ischemia, as low as 48% even in experienced hands (8). In a child without a surgical history, the initial ultrasound findings would have strongly supported a non-surgical diagnosis and likely led to discharge. It is important to recognize, though, that ultrasound has inherent diagnostic limitations in this setting, particularly when bowel gas artifact is pronounced, as it was in our patient. The ultrasound report should explicitly acknowledge these limitations rather than being interpreted as reassuring. However, non-surgical etiologies are not the only alternative in this age group. Intussusception typically affects children aged 3 months to 5 years and most commonly presents with intermittent abdominal pain, though the classic triad is uncommon, and younger patients may present atypically with lethargy (9). Perforated appendicitis can also cause mechanical small-bowel obstruction through inflammatory adhesions that kink or entrap bowel loops, and its overlapping features with gastroenteritis frequently delay diagnosis (10). In our patient, these entities were considered but excluded: intussusception by CT, which showed a clear adhesive transition point without a bowel-within-bowel configuration, and perforated appendicitis by the absence of an appendiceal mass on CT, non-visualization of the appendix on ultrasound, and intraoperative findings of pure adhesive obstruction without appendiceal pathology. The Bologna guidelines specifically state that when the adhesive etiology of obstruction is unsure, or when contraindications for non-operative management might be present, CT is the diagnostic technique of choice (8). This case confirms the importance of maintaining a low threshold for CT in children with surgical histories, even when initial ultrasound findings suggest non-surgical disease. Furthermore, Hyak et al. (2019) reported that in a cohort of 258 pediatric admissions for ASBO, there were no differences in leukocytosis or abdominal pain between patients who required urgent operation versus those managed non-operatively (11). This further illustrates that routine clinical and laboratory parameters cannot reliably distinguish children with ASBO who will require surgery from those who will resolve with non-operative management, reinforcing the diagnostic challenge highlighted in our case. Similarly, Earl et al. (2011) found that intestinal obstruction after pediatric liver transplantation was commonly related to unusual causes such as right-sided diaphragmatic hernia and post-transplantation lymphoproliferative disorder rather than adhesions, emphasizing the importance of maintaining a broad differential diagnosis in children with prior abdominal surgery (12).

Another important aspect of this case relates to the decision-making process between non-operative and operative management. The Bologna guidelines are explicit: non-operative management should always be tried in patients with ASBO, unless there are signs of peritonitis, strangulation, or bowel ischemia. Specifically, non-operative management is effective in approximately 70%–90% of patients with ASBO and includes nil per os, nasogastric decompression, and intravenous fluid and electrolyte supplementation (8). However, the evidence in children is more variable. A systematic review by Lin et al. (2014) examined conservative treatment for ASBO in children across seven studies and found that the success rate of non-operative management ranged from 16% to 75% (13). This wide range of success rates in the literature highlights the variability in patient selection and clinical presentation. In the present case, the patient did not have signs of peritonitis on initial examination (no guarding, no rebound tenderness, bowel sounds present), and she was hemodynamically stable. Therefore, a trial of non-operative management with admission for observation was entirely appropriate. However, the abdominal x-ray findings of small bowel dilatation to 3.5 cm, air-fluid levels, and an abrupt obstruction point raised sufficient concern that the surgical team was consulted, and CT was obtained without delay. The CT findings of pneumatosis intestinalis in the rectosigmoid colon, a finding that can precede perforation, tipped the balance toward urgent operative intervention. The Bologna guidelines note that delays in surgery increase morbidity and mortality (8). In this case, the decision to operate was made within 24 h of presentation, which was timely given the complete obstruction and pneumatosis. In contrast to the successful non-operative management seen in many adult patients, Eeson et al. (2010) found that 84% of children with ASBO eventually required surgery, differing markedly from results in adults where most resolve non-operatively (14). This finding aligns with our case, where surgical intervention was ultimately necessary.

Given this high rate of operative intervention in children, any adjunct that can help predict which patients will fail non-operative management is valuable. Water-soluble oral contrast is hyperosmolar and is thought to draw fluid from the bowel wall into the lumen, while serial imaging showing contrast beyond the small bowel indicates likely success of conservative management (15). In children, a recent systematic review and meta-analysis of 402 pediatric ASBO cases reported that water-soluble contrast had a pooled sensitivity of 98.9% and specificity of 83.1% for predicting progression to surgery, with no major contrast-related complications such as aspiration, anaphylaxis, or renal failure (16). However, that review found no significant difference in progression to surgery between children receiving water-soluble contrast-based non-operative management and those receiving standard non-operative management, and a separate meta-analysis of 11 randomized trials in adults also did not support a therapeutic benefit (15, 16). In our patient, CT confirmed complete obstruction with pneumatosis, so a contrast challenge was not attempted and surgery was appropriate.

The operative approach to ASBO in children is another important consideration. The Bologna guidelines acknowledge that laparoscopic surgery has been introduced in recent years and might decrease morbidity in subgroups of patients undergoing surgery for ASBO. However, the guidelines also caution that laparoscopy in an abdomen with very distended loops of bowel and multiple complex adhesions could increase the risk of severe complications such as enterotomies and delayed diagnosis of perforations, with reported bowel injury rates of 6.3% to 26.9% in laparoscopic cases. The guidelines recommend careful patient selection, noting that predictors for successful laparoscopic treatment include two or fewer prior laparotomies, appendectomy as the prior operation, no previous median laparotomy incision, and a single adhesive band (8). Consistent with this cautious approach, Mattone et al. (2018) reported a successful case of laparoscopic adhesiolysis in a 13-year-old child with ASBO, but noted that this approach is rarely performed in children and that patient selection is critical (17). In contrast, Choudhry and Grant (2006) found in their retrospective study of 414 neonates who underwent laparotomy that a single band caused obstruction in 35% of cases, multiple adhesions in 26%, and dense adhesions in 39%, indicating that many children present with complex adhesive disease not suitable for a simple laparoscopic approach (18). In the present case, the patient had multiple prior laparotomies, including NEC resection with jejunostomy creation and subsequent stoma closure. The operating surgeon appropriately chose an open laparotomy approach, which allowed for thorough exploration and safe adhesiolysis without enterotomy or other complications.

A unique aspect of this case is the postoperative identification of three concurrent infections from three different pathogen classes. The co-occurrence of bacterial, viral, and parasitic infections in a previously stable postoperative child is exceptionally rare. The ESBL E. coli urinary tract infection likely represented a healthcare-associated infection. Such organisms are increasingly prevalent worldwide and are resistant to most penicillins, cephalosporins, and aztreonam, necessitating carbapenem therapy such as meropenem. The adenovirus infection likely explained the mild upper respiratory symptoms noted prior to admission (runny nose one week earlier) and may have contributed to the mesenteric lymphadenopathy seen on the initial ultrasound. The chest x-ray findings of bilateral granular opacities and mild pleural effusion, combined with the positive adenovirus respiratory panel, were likely due to adenovirus infection. The Entamoeba histolytica/dispar finding is more difficult to interpret, as the two species are morphologically identical but only E. histolytica causes invasive amebiasis. The presence of 7 pus cells per high-power field and 2 + mucus in the stool suggests colonic inflammation that could be consistent with pathogenic amebiasis, but it could also be explained by the other infections or postoperative changes. Beyond the infectious complications of ASBO, the condition itself can have serious outcomes. While our case involved three concurrent infections in the postoperative period, van den Bunder et al. (2023) reported a fatal outcome in a 12-year-old boy with ASBO following pyloromyotomy during infancy, highlighting that ASBO can have life-threatening consequences if not recognized promptly (19).

Lastly, this case highlights the special considerations for young patients with ASBO. The Bologna guidelines include a specific section on “young patients,” noting that the risk of adhesion-related complications is lifelong and that pediatric patients have a particularly high risk (8). In a large cohort study of 898 infants who underwent laparotomy, Fredriksson et al. (2016) reported a 12.6% incidence of ASBO requiring surgery over a median follow-up of 14.7 years, with necrotizing enterocolitis being among the highest-risk diagnoses (25% incidence). Notably, 30% of ASBO episodes occurred after two years from the initial surgery, consistent with our patient who presented three years after her neonatal procedure (5). This pattern is consistent with published data: Choudhry and Grant (2006) found that although the majority of adhesions (87%) occur within one year of neonatal laparotomy, NEC is among the highest-risk conditions for subsequent ASBO (18). Similarly, Han et al. (2025) described a 7-month-old ex-premature infant (24 weeks gestation, 640 g) with a history of small bowel perforation and ileostomy closure who presented with bilious vomiting and abdominal distension due to adhesive small bowel obstruction requiring adhesiolysis, further illustrating the vulnerability of ex-premature infants to this complication (20). The present case reinforces that ASBO can occur years after the index surgery and that young patients remain at risk throughout childhood and adolescence. Clinicians caring for such patients should counsel families about the signs and symptoms of ASBO and the importance of seeking prompt medical attention for abdominal pain, vomiting, or constipation. Furthermore, because young patients have the highest lifetime risk of recurrence, they may benefit from adhesion prevention strategies such as minimally invasive surgical techniques and adhesion barriers, as noted in the Bologna guidelines (8).

### Strengths and limitations

The strengths of this case report include the detailed documentation of the patient's clinical course from initial presentation through surgery and postoperative recovery, including imaging and laboratory data from multiple time points. The availability of prior medical records from the referring institution provides important context about the patient's neonatal history and confirms the details of her NEC diagnosis and surgical management. The case also has limitations. The lack of species differentiation for Entamoeba histolytica versus dispar means that we cannot definitively state whether the patient had true amebiasis requiring treatment or non-pathogenic colonization. Additionally, while the viral panel identified adenovirus, no viral load or serotyping was performed, limiting our understanding of the clinical significance of this finding. The patient was discharged and lost to follow-up, so long-term outcomes including any recurrence of ASBO are unknown. Furthermore, the exact indication for starting meropenem and metronidazole on postoperative day 2, before the fever on day 17, is not explicitly documented in the available notes.

## Conclusion

In summary, children with prior abdominal surgery remain at lifelong risk for adhesive small bowel obstruction, normal ultrasound does not exclude this diagnosis, and postoperative fever may result from multiple concurrent pathogens.

### Implications for clinical practice

Clinicians should obtain CT without delay when adhesive small bowel obstruction is suspected in a child with prior abdominal surgery, even if ultrasound shows only reactive lymph nodes or fecal loading. For patients managed non-operatively, a 72-hour trial is safe per the Bologna guidelines, but failure to improve within this window should prompt surgical consultation. In patients with multiple prior laparotomies and anticipated complex adhesions, open laparotomy may be preferable to laparoscopy. At discharge, counsel families on warning signs of recurrent ASBO, such as colicky abdominal pain, bilious vomiting, abdominal distension, with clear instructions to return for evaluation rather than attempting home management.

## Data Availability

The original contributions presented in the study are included in the article/Supplementary Material, further inquiries can be directed to the corresponding author.
